# Supplementary material for: Salivary Immunoglobulin Gamma-3 Chain C Is a Promising Noninvasive Biomarker for Systemic Lupus Erythematosus
Source: Int J Mol Sci. 2021 Jan 29;22(3):1374. doi: 10.3390/ijms22031374 (PMC7866502; doi:10.3390/ijms22031374)
Supplement: Supplementary file 1 [file ijms-22-01374-s001.pdf]

Supplementary Table S1. Clinical characteristics of the subjects in 2-dimensional gel electrophoresis.

	SLE	HC
Number	11	11
Age, years	35.0 (29.0 – 44.0)	32.0 (30.0 – 40.0)
Sex (F/M)	11/0	11/0
Leukocyte, /μL	4400 (3825 – 7575)	
Lymphocyte, /μL	1402.5 (730.3 – 1922.3)	
Platelet, x 10^3/μL	262.0 (214.3 – 342.8)	
ESR, mm/h	32.5 (6.8 – 46.5)	
Low Complement level, n	8/11	
Anti-dsDNA Ab (+), n	7/11	
Mucocutaneous involvement, n	9/11	
Arthritis, n	5/11	
Nephritis, n	4/11	
Hematologic involvement, n	3/11	
SLEDAI	8 (8 – 9.3)	

SLE, systemic lupus erythematosus; HC, healthy control; ESR, erythrocyte sedimentation rate; dsDNA, double-strand DNA; Ab, antibody; SLEDAI, systemic lupus erythematosus disease activity index.

Supplementary Table S2. Clinical characteristics of the subjects in western blot analysis.

	SLE	RA	HC
Number	12	8	8
Age, years	39.4 (33.5 – 44.8)	40.5 (36.5 – 44.0)	35.0 (29.3 – 41.5)
Sex (F/M)	12/0	8/0	8/0
Leukocyte, /μL	3900.0 (2725.0 – 5700.0)		
Neutrophil, /μL	2452.9 (1195.1 – 3525.4)		
Lymphocyte, /μL	1064.0 (882.0 – 1323.0)		
Platelet, x 10^3/μL	202.0 (158.0 – 225.0)		
ESR, mm/h	8.5 (7.5 – 14.0)		
Low Complement level, n	8/12		
Anti-dsDNA Ab (+), n	6/12		
Mucocutaneous involvement, n (%)	5/12		
Arthritis, n (%)	6/12		
Nephritis, n (%)	6/12		
Hematologic involvement, n (%)	4/12		
SLEDAI	4.5 (4.0 – 6.0)		

SLE, systemic lupus erythematosus; RA, rheumatoid arthritis; HC, healthy control; ESR, erythrocyte sedimentation rate; dsDNA, double-strand DNA; Ab, antibody; SLEDAI, systemic lupus erythematosus disease activity index.
